# Supplementary material for: Current knowledge of vector-borne zoonotic pathogens in Zambia: A clarion call to scaling-up “One Health” research in the wake of emerging and re-emerging infectious diseases
Source: PLoS Negl Trop Dis. 2022 Feb 4;16(2):e0010193. doi: 10.1371/journal.pntd.0010193 (PMC8849493; doi:10.1371/journal.pntd.0010193)
Supplement: Attachment — Submitted filename: Response_to_Reviewers.docx [file pntd.0010193.s001.docx]

**Responses to Reviewers’ comments**

**Reviewer #1**: I have no methodological concerns. The authors execute an appropriately designed scoping review to achieve the stated objective of identifying research gaps for vector-borne zoonotic pathogens in Zambia.

***Response****: We thank the reviewer for their supportive comment.*
 **Reviewer #2**: As this was a scoping review article, many of the methodology criteria do not apply. However, in areas where the methods are outlined (lines 150-164), it would be nice to see how many articles were identified, and then which exclusionary filters were imposed. Including the number of studies that were removed at each step would also be ideal. For instance, "articles in languages other than English, n = __". Doing so would also provide more evidence that VBD are understudied in Zambia.

***Response:*** *We thank the reviewer for this suggestion. However, we are of the view that implementing all the suggested methods would make the manuscript read more like a systematic review. Since the general purpose of this scoping review was to identify the available literature and determine the knowledge gaps on vector-borne pathogens in Zambia, we have revised the methods section so as to indicate the total number of articles identified and included in this review (Lines 156.164)*

**Reviewer #3**: -The objectives of the study was clearly articulated a with the study design appropriately addressing the stated objectives. Adequate literature was reviewed to make up sufficient sample size to ensure adequate power to address the hypothesis being tested.

***Response:*** *We thank the reviewer for the encouraging remarks.*
 --------------------
**Results
-Does the analysis presented match the analysis plan?
-Are the results clearly and completely presented?
-Are the figures (Tables, Images) of sufficient quality for clarity?**

**Reviewer #1**: Lines 250-254: “Recently, a study conducted in 2016 among residents of Lukanga swamps in Central Province, revealed a 36.9% (79/214) sero-positivity for CHIKV and 19.6% (42/214) for Mayaro virus (MAYV) [24]. A recent study that screened for alphaviruses using 9,699 mosquitoes collected in Zambia (2014-2017) could not detect known alphaviruses (CHIKV, O'nyong'nyong virus, SINV etc.) [37].” Taken together, these results are quite striking – the two studies report essentially opposite findings. The authors should provide additional commentary on the research gap that is highlighted by these contrasting results, and propose hypotheses as to why such high seropositivity is observed near Lukanga, while the collection of nearly 10,000 mosquitos in Zambia resulted in no alphavirus detection.

***Response****: To help clarify the contrasting views of the two studies, we have given additional commentary in the results section clarifying that the study screening for CHIKV in mosquitoes did not include Lukanga swamps as one of the study areas. Additionally, whilst a huge number of mosquitos were screened, the majority were culex mosquitoes which are not the principal vectors for CHIKV. This compelled us to make a call for future studies to include Lukanga swamps as a potential good site to study alphaviruses and to explore use of mosquito trapping techniques that could maximize the capture of Aedes mosquitoes which are the primary vectors for CHIKV and MAYV (Lines: 250-260)*

Reviewer #2: Would recommend reformatting of Tables 1 & 2, which I outline in more detail within my general comments. Ultimately, generating some form of table or figure clearly showing how many studies were detected for each category (serologic vs. pathogen detection) and either stratifying on the basis of vector (as they have done) or pathogen type (virus, bacteria, parasite, etc) would be helpful. This would also highlight the lack of information available on pathogen detection as compared to serologic detection.

***Response****: Table 1 and 2 have been modified accordingly.*

**Reviewer #3**: The analysis presented match the analysis plan however a substantial part of the " Results" as presented was supposed to have been captured under "Discussion". From line 422 to the end should have been recommendation not results.

***Response****:* *We thank the reviewer for this suggestion. It is our considered opinion that this section highlights the research gaps on vector-borne zoonotic pathogens in Zambia as identified from the articles reviewed, and as such, should be treated as results of the scoping review. However, we have now deleted the first sentence (423-424) of the queried section that is giving the impression that this concerned section contains recommendations as opposed to results. The remaining parts under research gaps are there to give context with regards to the identified research gaps. In general, sentences in the results section that were discussing or giving detailed interpretation of the results have been moved to the discussion section as suggested by the Reviewer.*

--------------------
**Conclusions
-Are the conclusions supported by the data presented?
-Are the limitations of analysis clearly described?
-Do the authors discuss how these data can be helpful to advance our understanding of the topic under study?
-Is public health relevance addressed?**

**Reviewer #**1: Lines 427-429: The authors correctly highlight a weakness of the existing literature for Zambia, that “the majority of the studies were serological in nature.” Particularly because of their focus on arboviruses, the authors should highlight the extensive and well-documented cross-reactivity of antibodies between flaviviruses of different species. This represents a major caveat for interpreting any epidemiological studies that rely solely on flaviviral serosurvey data. The poor specificity of flaviviral serologic tests makes it quite challenging to derive accurate estimates of disease attributable to specific flaviviral species. Highlighting this methodological weakness of serosurveys further underscores the authors’ call “for increased genomic surveillance” (Line 430) as a complementary experimental approach.

In the present manuscript, there is no discussion of vector control efforts to combat zoonoses in Zambia, however understanding Zambia’s history of vector control is necessary to contextualize the reviewed studies. To this end, the authors should include a brief discussion of major vector control policies and efforts/campaigns (insecticide spraying, etc.) that have taken place in Zambia and focused on zoonotic pathogens, particularly if there are studies that measured vector populations or disease incidence pre- and post- control interventions. (Even if there are no published studies, such data may be available from the Ministry of Health.) If there are no such studies, the authors should explicitly highlight this as a research gap.

***Response****: We thank the reviewer for these insightful suggestions. To this effect, we have included a discussion point (Lines: 477-493) to highlight the weakness of the reviewed serological data and that its interpretation should be done cautiously. We further made a call for increased genomic surveillance to support serological data for guiding policy in addressing the problem posed by vector-borne diseases in Zambia. Additionally, we have also added discussion points highlighting the lack of vector control policies aimed at combating vector-borne zoonoses in Zambia and the need to urgently develop some (Lines: 569-582)*

Reviewer #2: Yes, though as I outline in other parts of my review, I would like to see Tables 1 & 2 modified to highlight at higher resolution where the knowledge gaps lie, and perhaps use that more granular analysis to present some detailed/specific multi-pronged VBD surveillance strategies. Including some preliminary policy recommendations, or at least identifying funding priorities, would be helpful to begin actualizing or making tangible some of the actions that could be taken to enhance our understanding of VBD dynamics in Zambia.

***Response****: Table 1 and 2 have been modified accordingly.*

**Reviewer #3**: The study did not caption "conclusions" notwithstanding some of the findings of the study were supported by the data presented while some were not. Example of the not part of the study: The conclusion that the sparse capacity for diagnosis of vector borne pathogens is as a result of Zambian healthcare facilities being very limited, if any exist at all. The limitations of analysis were not clearly described.

***Response****: We have now delineated the parts of the discussion that are actually conclusions of the study and have been placed under a new heading –‘Conclusions’. Further, we have rephrased part of the conclusions to clearly highlight the potential limitation of the study (Lines: 596-619).*
 --------------------

**Editorial and Data Presentation Modifications**?
-**Use this section for editorial suggestions as well as relatively minor
-modifications of existing data that would enhance clarity. If the only modifications needed are minor and/or editorial, you may wish to
- recommend “Minor Revision” or “Accept”.**

**Reviewer #1:** None.

***Response:*** *NA*

**Reviewer #2:** Recommend reformatting Tables 1 & 2; please see Summary & General Comments. Are all papers that were reviewed cited somewhere in the paper? Rather, is it possible to find all papers covered within this scoping review within the references list?

***Response:*** *Table 1 and 2 have been modified as recommended. Yes, all the papers we have cited appear in the reference list.*

**Reviewer #3**: (No Response)

***Response:*** *NA*

--------------------

**Summary and General Comments**
-**Use this section to provide overall comments, discuss strengths/weaknesses of the study, novelty, significance, general execution and scholarship. You may also include additional comments forthe author, including concerns about dual publication, research ethics, or publication ethics. If requesting major revision, please articulate the new experiments that are needed.**

**Reviewer #1**: This manuscript by Mubemba et al. is a scoping review of vector-borne zoonotic pathogens in Zambia, with a focus on pathogens transmitted by mosquitos (particularly arboviruses), ticks, fleas, and tsetse flies. Overall, this is a strong, focused manuscript that achieves its stated objective of highlighting research gaps for vector-borne zoonoses in Zambia. The review also highlights some interesting emerging research areas specific to Zambia, particularly Q fever, Yersinia pestis, and multiple pathogens that were discovered in Zambia. As such, this manuscript provides an important foundation for setting research and public health priorities for these pathogens in Zambia and in sub-Saharan Africa more broadly. There are some minor typographical and grammatical errors that will need to be corrected before final publication, however, overall, the manuscript covers a diverse array of studies while summarizing them clearly and succinctly. My critiques are minor and focus on areas of the Discussion that should be expanded upon by the authors to emphasize research gaps, in support their main thesis.

I recommend Minor Revisions before the manuscript is accepted for publication.

***Response:*** *We thank the reviewer for this comment and for accepting to review our manuscript. Indeed, the recommendations of the reviewer have helped to improve the clarity of the manuscript.*

*We have now corrected typographical and grammatical errors in the manuscript and we also expanded on the discussion to include the appreciation of the weakness of flaviviruses serological studies (Lines: 477-493) that were the majority of the studies reviewed and the potential limitation they imposed on the study. We have further added discussion points on the lack of data on vector control policies highlighting the need for implementation of some vector control strategies (Lines: 569-582). As recommended by other reviewers, we have also moved portions of the results section that were detailing the results and blended them in the discussion section where possible to improve on clarity of the manuscript.*

**Reviewer #2**: This scoping review examined current knowledge and identified research gaps of vector-borne zoonotic pathogens in Zambia. The authors used several major scientific databases and a selected set of search terms to identify the presence of articles on this topic. Major findings included that there was a lack of representation of articles describing infection by means of pathogen isolation or detection of nucleic acid. Conversely, most articles to date have provided information on serological evidence of infection. Serological evidence is useful for identifying past exposure, but these data are limited in their interpretation. The authors have made good note of this, as they state that human burden of disease is difficult to attribute to any of these pathogens when the only information we have on host-pathogen interaction is seroconversion/evidence of exposure. Knowledge gaps in this area make implementation of national policy and advocating for transmission control funding difficult.

The authors have compiled a compelling body of work, highlighting the major knowledge gaps surrounding vector-borne disease circulation in Zambia. I really enjoyed reading this piece and agree with the authors on many points. A few areas worth building out/commenting on: Related to flaviviruses, there is a high degree of serologic cross-reactivity, so by pinpointing that the majority of what we know about mosquito-borne flavivirus circulation in Zambia is based on serologic evidence is concerning for many reasons - one being that in general, flavivirus serology is not very reliable (for many cases, and this is dependent upon the diagnostic platform being used).

I would recommend re-formatting Table 1 to highlight which of the studies indicate serologic evidence of infection and which describe genomic detection. You might consider having a darkened circle for genomic detection, open circle for serologic, and half-filled circle for both. You could even list the reference numbers in the box alongside the circle, which would allow for easier navigation to those articles of interest by the reader, and also eliminate the fifth column in the table.

 I appreciate the suggestions for increasing genomic surveillance. This portion of the discussion could be built out a bit more with specific suggestions for how to do this in a cost-effective manner. For instance, using banked samples (for instance, does Zambia National Blood Transfusion Service (ZNBTS) have banked sera?) and consensus screening tools (pan-flavivirus, for instance) to maximize detection rates. Further, you might recommend increasing genomic surveillance not only in vectors and reservoir hosts, but in companion animals and domestic livestock that share habitat and interact frequently with humans - essentially, using them as sentinels for arboviral circulation in human populations. While there are limitations to this approach with regard to arthropod bloodmeal/host preference, this would allow for an analysis into which pathogens are being transmitted by arthropods in the same geographic region.

I would also build out Table 2 in a way that stratifies the suggestions. You could stratify each row by capacity-building, diagnostics, and surveillance… and then make recommendations for priority funding areas if that is possible. You might also consider stratifying each row on the basis of geographic regions of coverage… so for instance, “hospital/focal surveillance”, “regional surveillance”, or more integrated national surveillance programs to track pathogen spread and evolution. Another stratification could be “immediate”, “short-term”, and “long-term”, where more immediate recommendations might include some of the education/awareness campaigns you outline in the discussion. These are just a few ideas, and I suspect you could do this in a number **of different ways to help drive home the impact of these** recommendations. Table 2 is really the big take-home from this paper and could be featured in a way that allows for strong identification of patterns and then building off of that, some solid policy recommendations, or at least identification of funding priorities.

***Response:*** *We thank the reviewer for these perceptive comments and suggestions and for accepting to review our manuscript. Indeed, the recommendations of the reviewer have helped to improve the clarity of the manuscript. As recommended, we have expanded the discussion building on what genomic surveillance approaches could be employed. We agree with the reviewer’s suggested approaches as they are not only cost effective but also offer alternative and precise guiding tools in establishing the actual disease burden arising from arboviral infections in Zambia. Additionally, Table 1 and 2 have been modified accordingly as recommended (Lines 483-492).*

**Reviewer #3**: -Why was the focus only on mosquitoes, ticks, fleas and tsetsefly as vectors and not others such as blackfly etc

***Response:*** *We are of the opinion that the vectors we selected for the preparation of this review transmit major emerging and re-emerging pathogens of public significance and at least, some of these pathogens have been reported in Zambia. Besides, ticks and mosquito vectors transmit a significant number of emerging and re-emerging pathogens the world is battling with (Kilpatrick AM, Randolph SE. Drivers, dynamics, and control of emerging vector-borne zoonotic diseases. Lancet. 2012;380(9857):1946-1955. doi:10.1016/S0140-6736(12)61151-9).*

- Line 126 to 129 should be referenced otherwise it is a serious assertion to make if unsubstantiated

***Response:*** *We have inserted an appropriate reference for the sentence (**Asante J, Noreddin A, el Zowalaty ME. Systematic Review of Important Bacterial Zoonoses in Africa in the Last Decade in Light of the “One Health” Concept. Pathogens (Basel, Switzerland). 2019;8: 50. doi:10.3390/pathogens8020050)*

*.*

-On line 191, it is stated that Culex mosquito is a "suspected " but not a confirmed vector of NTAV. Which is the confirmed one then?
-Which other countries have been reported for NTAV, dengue, zika, WNV, Chikungunya, RVF, tick borne bacterial diseases and flea borne zoonotic pathogens as reported for yellow fever which is in 32 other tropical countries aside Zambia

***Response:*** *We thank the reviewer for this comment. To the best of our knowledge, no vector has been accurately been identified for NTAV. The study that suspected Culex mosquitoes made use of pools and the Culex mosquitoes were the majority in the pools that tested positive. Otherwise, more studies are needed to determine the actual vector. To be uniform and to avoid vagueness in our narrative, we have also deleted the sentence reporting the number of countries where yellow fever was reported and we hope this approach cures the query raised above.*

-When was the first case of encephalitis, zika and CCHF recorded in Zambia? Are there no mosquito borne bacteria infections' study in Zambia? Which of the infection in line 274 and 275 is line 276-277 describing-its symptoms? -What are the symptoms of borreliosis and Q-fever?

***Response:*** *To the best of our knowledge, no human cases of encephalitis, zika, CCHF and mosquito-borne bacterial infections have been recorded in Zambia. We have now specified the pathogen whose symptoms are listed between line 274-275. Further, we have also indicated the missing symptoms of both borreliosis and Q-fever in the manuscript text (Lines: 282-284; 295-296; 312-313).*

-Edit the name of the Table 1 by removing the first words which is ‘A summary table showing the number of' and add of Zambia after the 'humans' - A footer should be shown below Table 1 to show what ' X ‘signifies

***Response:*** *The correction to the heading of the table has been made. In addition, we have modified the table accordance with suggestions from other reviewers and the mark ‘X’ no longer appears in the table. However, we have still defined in the footnotes of the table what the ‘new contents’ of these columns mean.*

-Give reference to the sentence on line 465

***Response:*** *An appropriate reference has been inserted* *(Salyer SJ, Silver R, Simone K, Behravesh CB. Prioritizing zoonoses for global health capacity building—themes from one health zoonotic disease workshops in 7 countries, 2014–2016. Emerging Infectious Diseases. 2017;23: S57–S64. doi:10.3201/eid2313.170418)*

- On line 468, it is stated that '...should be targeted for both field and facility-based surveillance efforts' – remove the word 'effort'

***Response:*** *The correction has been made.*

- Give reference to the sentences in lines 474 to 476

***Response:*** *An appropriate reference has been inserted (Briand S, Beresniak A, Nguyen T, Yonli T, Duru G, Kambire C, et al. Assessment of yellow fever epidemic risk: An original multi-criteria modeling approach. PLoS Neglected Tropical Diseases. 2009;3. doi:10.1371/journal.pntd.0000483; Bhatt S, Gething PW, Brady OJ, Messina JP, Farlow AW, Moyes CL, et al. The global distribution and burden of dengue. Nature. 2013;496: 504–507. doi:10.1038/nature12060: Musso D, Gubler DJ. Zika virus. Clinical Microbiology Reviews. 2016;29: 487–524. doi:10.1128/CMR.00072-15 etc)*

- On line 576-578, the sentence is speculative and should be stated as such
 - Acknowledgments on line 586 is spelt wrongly

***Response:*** *The corrections have been made to indicate that this sentence was a considered opinion of the authors (Lines: 608-6119). In addition, the word “acknowledgements” has been spelt correctly now)*

-The 'results’ section had a lot of discussion which should be moved to the 'discussion' section. Rewrite your results and discussion.***Response:*** *We thank the reviewer for this suggestion. As indicated in earlier responses, we have now removed sentences in the results section detailing or suggesting the interpretation of the results. Where possible, we have blended these sentences in the discussion.*
