# Supplementary material for: Current knowledge of vector-borne zoonotic pathogens in Zambia: A clarion call to scaling-up “One Health” research in the wake of emerging and re-emerging infectious diseases
Source: PLoS Negl Trop Dis. 2022 Feb 4;16(2):e0010193. doi: 10.1371/journal.pntd.0010193 (PMC8849493; doi:10.1371/journal.pntd.0010193)
Supplement: Attachment — Submitted filename: Letter_Response_to_Reviewers.docx [file pntd.0010193.s002.docx]

**Macha Research Trust**

**doing business as**

**MALARIA INSTITUTE AT MACHA**

**P.O. Box 630166**

**Choma, Zambia**

**Phone/Fax: + 260 976551411**

**20^th^ January, 2022**

The Editorial Office

PLoS Neglected Tropical Diseases

Dear Editor,

We are grateful for the opportunity to revise the manuscript – “**Current knowledge of vector-borne zoonotic pathogens in Zambia: A clarion call to scaling-up “One Health” research in the wake of emerging and re-emerging infectious diseases**” We hope that the effected changes as suggested by reviewers have both helped clarify and strengthen the manuscript. Details regarding our specific responses to the reviewers’ suggestions are detailed in the subsequent pages.

Thank you for considering our manuscript for publication in your journal and we hope we have addressed the raised concerns adequately.

Yours Sincerely,

Edgar Simulundu (PhD)

**Corresponding Author**

**Responses to the Reviewers**

**Methods**
-Are the objectives of the study clearly articulated with a clear testable hypothesis stated?
-Is the study design appropriate to address the stated objectives?
-Is the population clearly described and appropriate for the hypothesis being tested?
-Is the sample size sufficient to ensure adequate power to address the hypothesis being tested?

-Were correct statistical analysis used to support conclusions?
  -Are there concerns about ethical or regulatory requirements being met?

**Reviewer #1**: No concerns.

***Response****: We thank the reviewer for their support*

**Reviewer #2**: Table 1 is much improved with the stratification of publications based on whether detection was serologic or molecular in nature.

***Response****: We thank the reviewer for their supportive comment*

**Reviewer #3**: (No Response)

***Response:*** *NA*

**Results**
-Does the analysis presented match the analysis plan?
-Are the results clearly and completely presented?
-Are the figures (Tables, Images) of sufficient quality for clarity?

**Reviewer #1**: No concerns.

***Response****: We thank the reviewer for their support*

**Reviewer #2**: It is striking to me how unevenly distributed Table 1 is. Look at the number of papers providing evidence of exposure to mosquito-borne arboviruses in humans compared to parallel data available on animals and vectors. It appears that surveillance of mosquito-borne viruses in vectors and animals is hugely lacking and those data are critical when considering these transmission cycles through a One Health lens. Conversely, there are MANY papers demonstrating molecular detection of bacterial/protozoal tick-borne, flea-borne, and tsetse-borne diseases in animals and arthropod vectors, and to a lesser extent in humans.

***Response****: Indeed, we agree with the observations of the Reviewer and it is for that reason that we made a clarion call for increased genomic surveillance of arboviral pathogens in humans and we have made suggestions on how that could be implemented to complement the existing serological data (Lines 477-493). In addition, we also made very specific research and diagnostic priorities (Table 2) to guide on how future research activities could be implemented so that we have a balanced research approach in informing One Health strategies for the country. Once properly implemented as suggested in Table 2, we foresee a robust formulation of national policies that could help in the control and mitigation of the impact of these diseases in Zambia and further enable the country to prepare for future epidemics, including pandemics.*

**Reviewer #3**: (No Response)

***Response****: NA*

**Conclusions**
-Are the conclusions supported by the data presented?
-Are the limitations of analysis clearly described?
-Do the authors discuss how these data can be helpful to advance our understanding of the topic under study?
-Is public health relevance addressed?

**Reviewer #1**: No concerns.

***Response****: We thank the reviewer for their support.*

**Reviewer #2**: I think discussing how targeting only one aspect of the One Health triad (or in this case, one aspect of the transmission cycle) risks over-interpretation of the role that component plays in the cycle, and the general imbalance between studies suggests that this pattern may translate to our overall understanding of these processes on a larger scale. I see now that you’ve included in the beginning of the conclusions that it’s problematic that a majority of human-focused studies contained only serologic evidence, complicating the interpretation of this data (or ability to associate it with disease burden, levels of shedding/viremia, etc.) However, I think it’s worth elaborating on the point you make in lines 146-149. WHY is there such an imbalance in molecular detections? Sampling bias?

***Response****: We thank the reviewer for this suggestion. The reason for the imbalance in molecular detections is not clear, but we suspect a lack of laboratory capacity, particularly in rural healthcare centres where vector-borne zoonotic diseases are most likely to emerge. To further elaborate on the need to stimulate research on* *emerging and re-emerging zoonotic pathogens in Zambia, we have now stated that genomic surveillance is still hugely lacking even for pathogens for which serological data exists and hence, there is an urgent need to invest in genomic surveillance if the country is to make positive strides in guiding the response, diagnosis and management of zoonotic diseases (Lines 147-151).*

**Reviewer #3**: (No Response)

***Response****: NA*

**Editorial and Data Presentation Modifications**
Use this section for editorial suggestions as well as relatively minor modifications of existing data that would enhance clarity. If the only modifications needed are minor and/or editorial, you may wish to recommend “Minor Revision” or “Accept”.

**Reviewer #1**: No concerns.

***Response****: We thank the reviewer for their support.*

**Reviewer #2**: Line items:

On page 19 of document, lines 419-422, it appears something happened with the ‘track changes’, as I believe the last sentence of that section is fragmented.

***Response****: We thank the reviewer for this observation. We have now reconstructed the sentence by cutting it into two and rephrasing sections of it (Lines 408-409)*.

Missing reference in page 2 of the discussion, and page 6 (line 118, 121) of discussion, Line 123: ‘one-health’ is used but in the rest of the paper, you use One-Health (capitalized). Also, I think I’ve seen it used more often in the past without a hyphen, but I’ll defer to the editor on that.

***Response****: We apologise for the mix-up between the tracked and the cleaned versions of the manuscript. In the tracked version, the references were indeed missing but we do confirm that they appear in the cleaned version as references 98 and 98 (Lines 488-489). In addition, we have now replaced the word “One-Health” with one without a hyphen wherever it appeared and we have also used the capitalised one (Lines 77, 100, 585, 622)*

 There were some comments in the conclusions (lines 144-145) that I found a bit confusing.

***Response****: The queried sentence was stated in the context of possible limitations of the study. We note that serological studies which were the majority of the studies reviewed, do not in themselves give a true picture of the disease burden particularly for arboviruses as these serological assays are prone to cross-reactivity.*

Reviewer #3: (No Response)

***Response****: NA*

**Summary and General Comments**
Use this section to provide overall comments, discuss strengths/weaknesses of the study, novelty, significance, general execution and scholarship. You may also include additional comments for the author, including concerns about dual publication, research ethics, or publication ethics. If requesting major revision, please articulate the new experiments that are needed.

**Reviewer #1**: This manuscript by Mubemba et al. is a scoping review of vector-borne zoonotic pathogens in Zambia, with a focus on pathogens transmitted by mosquitos (particularly arboviruses), ticks, fleas, and tsetse flies. This work provides an important foundation for setting research and public health priorities for these pathogens in Zambia and in sub-Saharan Africa more broadly. While there remain some minor typographical and grammatical errors that will require editing before final publication, in terms of content, the authors have fully addressed all my critiques. Indeed, the authors' revisions to Table 1 (based on the suggestions of Reviewer #2) have markedly improved the clarity and visual presentation of the literature review. I therefore support acceptance of the manuscript for publication.

***Response****: We thank the reviewer for their support of our manuscript and for accepting to review it. Their insightful suggestions significantly helped to improve the quality of the manuscript.*

**Reviewer #2**: (No Response)

***Response:*** *NA*

**Reviewer #3**: All scientific names should be italized

***Response:*** *We thank the reviewer for their support of our manuscript and for accepting to review it. We have now proof read the manuscript ensuring that all scientific names are italicised.*
